# Exists a role for serum irisin in Egyptian Behcet’s patients with subclinical atherosclerosis?

**DOI:** 10.1007/s10067-022-06368-9

**Published:** 2022-09-16

**Authors:** Mohamed A. Ismail, Ola Mounir, Ahmed Sedky, Hisham A. Algahlan, Esam A. Abda, Ahmed R. Radwan, Hanan Sayed Abozaid

**Affiliations:** 1 Department of Rheumatology and Rehabilitation, Sohag University Hospital, 82524 Sohag, Egypt; 2 Department of Clinical Pathology, Sohag University, Sohag, Egypt; 3 Department of Diagnostic Radiology, Sohag University, Sohag, Egypt; 4 Department of Rheumatology and Rehabilitation, Assuit University, Asyut, Egypt

**Keywords:** Atherosclerosis, Egyptian Behcet’s disease, Serum irisin

## Abstract

**Objectives:**

To examine the serum irisin level in a group of Behcet’s disease patients, its association with illness parameters, and its utility in diagnosing subclinical atherosclerosis.

**Methods:**

This randomized case–control study included 50 patients and 50 age- and sex-matched controls. Carotid Doppler ultrasound for the measurement of the carotid artery intima-media thickness (CIMT) and ankle-brachial pressure index (ABPI) were performed. A clinical evaluation, lipogram, and serum irisin were also performed.

**Results:**

Between the patients and the control group, there was a significant difference in CIMT, S. irisin level, and ankle-brachial pressure index; however, gender and BMI did not significantly affect CIMT, ABPI, or S. irisin level. CIMT demonstrated a substantial negative correlation with both S. irisin and ABPI (*r* =  − 0.62, *P* 0.0001).

With a sensitivity of up to 94.30% and a specificity of 93.30%, the ROC analysis revealed that a decrease in S. irisin level in Behcet’s patients was indicative of subclinical atherosclerosis. The drop in the ABPI level demonstrated a sensitivity of up to 94.30% and a specificity of 100%.

**Conclusion:**

Subclinical atherosclerosis is prevalent among Egyptian Behcet’s patients, and S. irisin can be employed as a biomarker for diagnosing subclinical atherosclerosis in Behcet’s illness.

**Table:** 

**Key Points** • *Serum irisin has been studied in numerous autoimmune disorders as a marker for subclinical atherosclerosis, although its importance in Behcet’s disease remains unclear (BD)*. • *We examined the change in serum irisin levels in Behcet’s disease patients and healthy controls. In addition, its association with carotid artery intima-media thickness (CIMT) and ankle-brachial pressure index was investigated (ABPI)*. • *Changes in serum irisin levels are significant in BD, and a decrease in irisin level indicates subclinical atherosclerosis*.	

## Introduction

Behcet’s disease (BD) is a chronic, relapsing, inflammatory disorder characterized by systemic involvement, vascular damage, and endothelial cell dysfunction [1, 2].

Cardiovascular involvement ranges from 7 to 46% [1, 3], and atherosclerotic heart disease with myocardial infarction is the leading cause of death in BD patients [4].

Chronic inflammatory disorders are characterized by systemic inflammation that leads to cardiovascular disease (CVD) by well-established mechanisms: accelerated atherosclerosis, insulin resistance (IR), platelet dysfunction, hypercoagulability, hypercholesterolemia, and hyperglycemia [5].

Endothelial dysfunction is a well-recognized indicator of subclinical vascular atherosclerosis; it is determined by the intima-media thickness of the common carotid artery (CIMT).

Endothelial dysfunction is the first manifestation of BD’s vascular problems. It greatly contributes to the onset and progression of vascular damage in many body locations, resulting in metabolic disease consequences [6].

Vasculitis and inflammations of the artery wall can increase platelet aggregation and inhibit fibrinolysis, resulting in thrombosis [7].

Subclinical atherosclerosis is an early sign of atherosclerotic load, and its prompt diagnosis can postpone or prevent the development of overt cardiovascular disease [8].

Irisin is a myokine secreted by the liver, kidney, heart, skeletal muscles, and skin in response to exercise [9]. Previous research studied its potential as a therapeutic target for endothelial dysfunction and metabolic diseases [10].

In 2015, Lee HJ et al. established the association between circulating irisin levels, endothelial dysfunctions, and subclinical atherosclerosis in non-diabetic adult patients by finding a substantial correlation between carotid atherosclerosis and serum irisin levels in dialysis patients [11].

Recently Altay et al. investigated the relationship between inflammation and irisin in serum samples from chronic spontaneous urticaria (CSU) patients, they observed lower irisin levels in the CSU group, and they raised a suggestion that a decrease in irisin levels may be decisive for CSU [12].

The purpose of this study is to determine the link between serum irisin level and subclinical atherosclerosis in patients with Behcet’s illness and to compare serum irisin to other modalities for diagnosing subclinical atherosclerosis and endothelial dysfunctions.

### Patients and procedures

It is a case–control study that included 50 Behcet’s patients diagnosed according to 2006 classification criteria (ICBD, 2006) [13, 14], and 50 healthy controls matched for age and sex; the study included patients with disease duration of more than 6 months and age at disease onset after the age of 16; we excluded cases with concomitant systemic diseases such as chronic obstructive pulmonary disease, coronary artery disease, cancer, thyroid function disorder, and hematologic disorders.

The Ethics Committee of our university’s medical school approved the study under the number IBR ♯ S20-154, and all participants provided written informed permission.

All subjects underwent a comprehensive clinical examination, and disease activity was assessed using the Behcet Syndrome Activity Score (BSAS) [15].

Each participant’s 10 ml of venous blood was drawn under aseptic conditions using sterile disposable gloves for laboratory examinations.

A: Routine investigations, liver function tests (LFT), kidney function tests (KFT), fasting blood glucose, lipid profile including cholesterol, triglyceride, high density lipoprotein (HDL), and low-density lipoprotein (LDL) utilizing the Cobas c311 Chemistry Analyser System (Roche Diagnostic GmbH, Indianopolis, IN, USA).

The B complete blood count was performed by CELL-DYN Abbott (Abbott Laboratories, Diagnostic Division IL, USA) and the ESR was performed using a westergren tube.

C: The serum irisin concentration was determined with an irisin ELISA kit and an ELISA Thermo Fisher, Scientific Multiscan EX Microplate Reader, OY, FI-O1621, Vantaa, Finland.

#### Evaluation procedure

Step 1: prepare all reagents, working standards, blanks, and samples according to the instructions in the preceding sections.
Step 2: refer to the assay layout sheet to calculate the number of wells to place any unused wells and desiccant back into the pouch, reseal the pouch, and store the wells at 4C.
Step 3: add 50 mL of the standard to the testing standard well, followed by 10 mL of the testing sample (sample final dilution is fivefold). Pipette the sample to the wells, avoiding the well walls as much as possible, and mix gently.
Step 4: cover with the included adhesive strip and incubate for 30 min at 37 °C.
Step 5: configure liquid, 30 times dilute wash solution with distilled water to configure liquid.
Step 6: washing, remove the adhesive strip, discard the liquid, and then pipette washing buffer into each well. Allow to stand for 30 s and then drain.
Step 7: add enzymes, pipette 50 l of HRP-conjugate reagent into each well, excluding the well designated as a blank.
Step 8: incubate, operation with four
Step 9: washing, using number 6
Step 10: add 50 ml of chromogen solution A and 50 ml of chromogen solution B to each well, avoiding light for 15 min at 37 °C.
Step 11: stop the reaction by pipetting 50 ml of stop solution into each well (the blue change to yellow).

Calculate the value of the blank well as zero. Read absorbance at 450 nm 15 min after pipetting stop solution.

### Diagnostic imaging techniques for atherosclerosis

A: Carotid Doppler ultrasound, for the measurement of carotid artery intima-media thickness (CIMT): CIMT is defined as a low-level echo gray band that does not project into the arterial lumen. CIMT was measured during the diastolic phase as the distance between the leading edge of the first and second echogenic lines of the far walls of the distal segment of the common carotid artery, the carotid bifurcation, and the internal using superficial multifrequency linear array transducer [5–8 MHz] with a sonographic apparatus [Aplio 500 Canon medical system, Japan]. CIMT tests were done on artery segments devoid of plaque. To eliminate examiner bias, all examinations and measures have been conducted by the same examiner. Age-dependent normal range is used as the reference for CIMT. The identification of a carotid atherosclerotic plaque is defined as a focal IMT of 50% or > 0.5 mm relative to the adjacent artery wall, or an absolute IMT of > 1.5 mm [16].

B: Evaluation of ankle/brachial BP index utilizing blood pressure cuff Doppler pulse volume recording (PVR) ultrasound. Grayscale duplex Doppler is used to map arterial disease and to solve problems.

The index is a ratio of the pressure in the highest ankle artery / the highest brachial artery.

- 1.0–1.4: normal
- 0.91–0.99 borderline
-  ≤ 0.9: abnormal (i.e., PAD)
- 0.4 to 0.9: moderate to mild PAD
-  < 0.4: indicative of severe PAD

### Analytical statistics

Quantitative data were expressed as the mean, standard deviation, median, and range. The student *t*-test was used to compare the means of two groups. When data distribution was not normal, the Mann–Whitney test was utilized. Using chi square, qualitative data were presented as numbers and percentages and compared. Using a Roc curve analysis, the optimal cutoff was determined. Also calculated were sensitivity, specificity, positive predicted value, and negative predictive value. Pearson’s test of correlation was employed to determine the relationship between continuous variables. Excel or the STATA application was used to create graphs. Less than 0.05 was considered significant for the *P* value.

## Results

The study comprised 50 BD patients and 50 healthy controls with a mean age of 30.2, a mean onset age of 28.6 years, and a mean disease duration of 1.64 years, with a range of 1 to 5 years. 12 cases were female (24%) while 38 cases were male (76.6%). Regarding BSAS score, Behcet’s illness patients had a mean score of 5.6, with a range of 3 to 9.

A total of 100% of the patients presented with oral ulcers, 88% with genital ulcers, 80% with skin lesions, 68% with ocular lesions, and 44% with uveitis. Vascular lesions in the form of (DVT, S.V.T thrombosis, cerebral infarction, or recurrent stroke) were present in 26% of the patients, and neurological deficits in 10%.

The variables having a significant difference between patients and healthy controls are listed in Table 1, but non-significant variables such as lipid profile, fasting glucose, blood urea, serum creatinine, and liver enzymes are omitted.

**Table 1:** Comparison of the main features between the patients and the healthy control

Variable	Behcet’s *N* = 50	Controls *N* = 50	*P* value
Age/years: mean ± SD Median (range)	30.24 ± 6.13 29 (16:40)	29.84 ± 4.97 30 (21:39)	0.72
Females Males	12 (24.00%) 38 (76.00%)	12 (24.00%) 38 (76.00%)	1.00
BMI: mean ± SD Median (range)	26.75 ± 2.13 27.10 (20.7:29.4)	26.81 ± 2.02 27.23 (21.6:29.7)	0.88
Hemoglobin (g/dl) WBCs (103/ul) Platelets	12.66 ± 1.12 6.7 ± 1.63 237.9 ± 69.09	13.24 ± 1.03 6.04 ± 1.44 286.36 ± 82.51	0.01 0.03 0.002
ESR (mm/h)	49.3 ± 13.45	9.9 ± 4.43	< 0.0001
S. irisin (ng/ml)	32.1 ± 8.34	51.48 ± 8.49	< 0.0001
Ankle brachial pressure index	0.75 ± 0.21	1.19 ± 0.13	< 0.0001
cIMT (mm)	0.80 ± 0.29	0.56 ± 0.25	< 0.0001
Plaque	5 (10%)	0	0.06
Atherosclerosis	35 (70%)	0	< 0.0001
Q-T dispersion			
No	50 (100%)	50 (100%)	

Mann–Whitney test, Student *t*-test. Statistically significant difference (*P* < 0.05)

*BMI*, body mass index; *cIMT*, carotid intima median thickness; *S. irisin*, serum irisin

A subgroup analysis based on gender and body mass index is shown in Table 2; this analysis revealed non-significant differences in CIMT, S. irisin, and ankle brachial pressure index levels between males and females, while the results of the effect of BMI among the studied subgroups revealed that the BMI did not differ significantly (*P* = 0.37) among the control, but in the patients with Behect’s disease, there was a significant increase of BMI in females.

**Table 2:** Subgroup analysis according to gender and BMI

Variables	Patients with Behcet’s disease			Controls		
	Females *N* = 12	Males *N* = 38	*P* value	Females *N* = 12	Males *N* = 38	*P* value
cIMT (mm)	0.69 ± 0.16 0.56:1.1	0.83 ± 0.32 0.5:1.5	0.17	0.55 ± 0.23 0.3:1.2	0.56 ± 0.26 0.3:1.2	0.73
S. irisin (ng/ml)	34.83 ± 9.08 22:51	31.24 ± 8.02 14:48	0.20	52.5 ± 9.06 44:71	51.16 ± 8.40 40:67	0.64
Ankle brachial pressure index	0.78 ± 0.20 0.5:1.1	0.74 ± 0.21 0.4:1.1	0.52	1.22 ± 0.15 0.95:1.4	1.18 ± 0.13 0.9:1.4	0.31
BMI ( kg/m^2^)	27.82 ± 2.19 21.96:29.41	26.41 ± 2.02 20.76:29.41	0.04	27.27 ± 1.65 23.72:29.73	26.66 ± 2.12 21.60:29.74	0.37
BMI (kg/m^2^)						0.43
Normal Overweight	1 (8.33%) 11 (91.67%)	6 (15.79%) 32 (84.21%)	1.00	1 (8.33%) 11 (91.67%)	8 (21.05%) 30 (78.95%)	
	BMI < 25 (kg/m2) *N *= 7	BMI > 25 (kg/m2) *N *= 43		BMI < 25 (kg/m2)	BMI > 25 (kg/m2)	
cIMT(mm)	0.74 ± 0.36 0.5:1.5	0.80 ± 0.28 0.5:1.5	0.20	0.47 ± 0.07 0.4:0.6	0.58 ± 0.27 0.3:1.2	0.30
S. irisin (ng/ml)	36.14 ± 10.12 24:49	31.44 ± 7.96 14:51	0.17	51.89 ± 8.33 42:65	51.39 ± 8.62 40:71	0.88
Ankle brachial pressure index	0.8 ± 0.25 0.4:1.1	0.74 ± 0.20 0.4:1.1	0.46	1.12 ± 0.13 0.95:1.3	1.20 ± 0.13 0.9:1.4	0.15

Mann–Whitney test. Student *t*-test. Fisher exact test

Statistically significant difference (*P* < 0.05)

Comparing S. irisin levels between BD patients with and without ocular lesions and vascular events revealed no significant differences (32.69.3, 30.96.1 *P* = 0.5) (32.0 6.67, 32.18.92 *P* = 0.8), whereas patients with neurological deficits had significantly lower S. irisin levels than those without neurological deficits (27.24.2, 32.6 8.5 *P* = 0.04).

CIMT did not distinguish between patients with and without ocular, vascular, or neurological impairments.

Tests of correlation (Table 3):

- CIMT demonstrated a substantial positive association with age, disease onset age, WBCs, and ESR, whereas it demonstrated a significant negative correlation with S. irisin and ankle brachial pressure index.
- S. irisin had a favorable correlation with platelet count and ankle brachial pressure index. A statistically significant inverse correlation with the CIMT
- Conversely, the ankle brachial pressure index had a strong negative correlation with CIMT.
  The comparison of S. irisin and ABPI between subclinical atherosclerosis and non-atherosclerosis patients with Behcets (Table 4):
- Meanwhile, our findings regarding the relationship between S. irisin, ankle brachial pressure index, and subclinical atherosclerosis in patients with Behcet’s illness revealed a significant difference in S. irisin levels (27.8 ng/ml, 42.13 ng/ml, *P* value 0.0001) between individuals with subclinical and non-subclinical atherosclerosis. A decrease in ankle brachial pressure index is also correlated with an increase in subclinical atherosclerosis, with a significant difference (0.64, *P* value 0.0001 for a difference of 1.0)

**Table 3:** The significant correlations among different variables

CIMT			
Variable	*r* value		*P* value
Age/year	0.32		0.02
Age at onset	0.27		0.06
WBCs (10^3^/ul)	0.29		0.04
ESR (mm/h)	0.39		0.006
S. irisin (ng/ml)	− 0.62		< 0.0001
Ankle brachial pressure index	− 0.62		< 0.0001
S. irisin			
Platelets (10^3^/ul)	0.30	0.03	
CIMT	− 0.62	< 0.0001	
Ankle brachial pressure index	0.71	< 0.0001	
Ankle brachial pressure index			
BSAS score	0.25	0.058	
S. irisin (ng/ml)	0.71	< 0.0001	
cIMT	− 0.62	< 0.0001	

Pearson correlation test. Statistically significant difference (*P* < 0.05)

**Table 4:** Comparison of S. irisin and ankle brachial pressure index between patients with subclinical and non-subclinical atherosclerosis

Variable	No subclinical atherosclerosis *N* = 15	Subclinical atherosclerosis *N* = 35	*P* value
S. irisin (ng/ml)			
Mean ± SD Range	42.13 ± 5.46 33:51	27.8 ± 4.95 14:40	< 0.0001
Ankle brachial pressure index			
Mean ± SD Range	1.0 ± 0.07 0.9:1.1	0.64 ± 0.15 0.4:1	< 0.0001

Student *t*-test. Statistically significant difference (*P* < 0.05)

### The analysis of regression

**Fig. 1:**
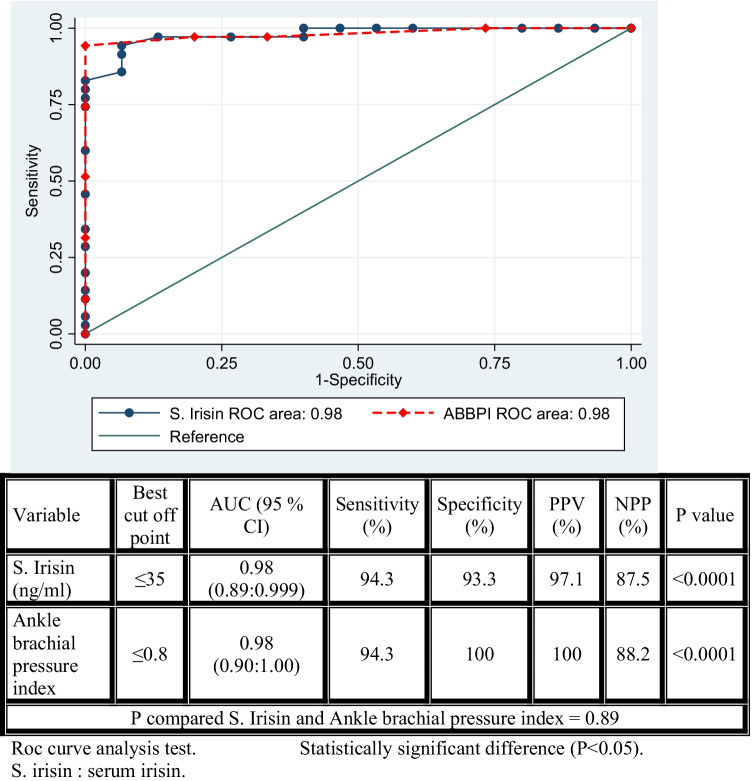
ROC analysis of S. irisin and ankle brachial pressure index in predicting subclinical atherosclerosis. Roc curve analysis test. Statistically significant difference (*P* < 0.05). S. irisin: serum irisin

- Univariate linear regression examination of factors influencing CIMT in Behect’s patients revealed a significant positive relationship with age (*P* = 0.03), ESR (*P* = 0.0006), and WBCs (*P* = 0.04), and a significant negative relationship with S. irisin (p0.0001) and ankle brachial pressure index (*P* 0.0001).
- Multivariate linear regression analysis of factors affecting CIMT in patients with Behcet’s disease (include significant variable in univariate) revealed that age of patient (*P* = 0.02) and ESR level (*P* = 0.01) were the most significant factors increasing incidence, while S. irisin (*P* = 0.04) and ankle brachial pressure index (*P* = 0.04) were the most significant factors decreasing incidence.
- The most important predictors of subclinical atherosclerosis in patients with Behcet’s illness are male gender, platelet count, S. irisin, and ankle brachial pressure index, as demonstrated in Table 5 of the multivariate logistic regression analysis of the components predicting subclinical atherosclerosis.
- The ROC analysis (Fig. 1) of S. irisin and ankle brachial pressure index in predicting subclinical atherosclerosis revealed that the decreased level of S. irisin in Behcet’s disease patients was indicative of subclinical atherosclerosis, with a sensitivity of 94.30% and a specificity of 93.30% (*P* 0.0001). The level of ankle brachial pressure index in patients with Behcet’s illness is also indicative of preclinical atherosclerosis, as its level reduced dramatically with a sensitivity of 94.30% and a specificity of 100% (*P* 0.0001), indicating subclinical atherosclerosis.

**Table 5:** Multivariate logistic regression analysis of factor predicting subclinical atherosclerosis in patient with Behcet’s disease include significant variable in univariate analysis

Variable	Odds ratio (95% confidence interval)	*P* value
Male gender	1.31 (0.07:23.39)	0.85
Platelets (10^3^/ul)	1.01 (0.98:1.03)	0.60
S. creatinine (mg/dl)	0.02 (0:66,859)	0.62
S. irisin (ng/ml)	0.55 (0.38:0.83)	0.004
Ankle brachial pressure index	0.0001 (0:0.003)	0.002

Multivariate logistic regression test. Statistical significant difference (*P* < 0.05)

## Discussion

Increased risk for cardiovascular lesions (CVD) in patients with pre-existing chronic inflammatory diseases at younger ages observed in some studies raised the assumption about endothelial dysfunction as a common initial lesion in the development of atherosclerosis [5, 17], and irisin, as a hormone-like myokine, Lee et al. demonstrated its association with endothelial dysfunctions and subclinical atherosclerosis in non-diabetic adult patients [11].

There are few studies examining S. irisin as a biomarker for diagnosing subclinical atherosclerosis in Behcet’s illness. In our study, we intended to investigate this topic.

We did not find a significant relationship between serum irisin level and disease activity score, nor did we find a relationship between CIMT and disease activity score. The BSAS score of the included patients had a mean value of 5.6, and a range of 3 to 9 indicates that they are in remission and have been on treatment for more than six months. These results concur with Icli et al.’s findings [18].

Subclinical atherosclerosis can begin early in life and be unnoticed until a cardiac infarction or stroke [19].

Hyperglycemia and hyperlipidaemia are known to accelerate the atherosclerotic process by direct endothelial dysfunction augmenting cytokine release with increasing lipid peroxidation and causing oxidative damage [20]; our results showed non-significant differences regarding fasting glucose, cholesterol, triglyceride, or HDL in patients and controls, which enabled us to estimate the relationship between irisin and atherosclerosis as there was no correlation.

There were no documented cases of Q-T dispersion; therefore, the atherosclerotic changes in our patients are asymptomatic and a sufficient narrowing of coronaries may be required for symptoms to appear; our results agreed with [21] Ross.1993 who demonstrated that atherosclerosis is asymptomatic for decades because the arteries enlarge at all plaque sites; consequently, there is no impact on blood flow.

Obesity is a condition characterized by excessive fat buildup and is frequently accompanied with insulin resistance (IR), type 2 diabetes (T2DM), and cardiovascular diseases (CVDs) [22, 23]. Our findings revealed a non-significant difference between whole patients and controls (*P*-value = 0.88); these findings were consistent with those of Koca et al. [24], who reported that the prevalence of obesity was not higher in the BD group than in the HC group or the general population.

The non-significant difference for ankle brachial pressure index between those of BMI 25 and those of BMI > 25 was cleared by the controlled blood glucose level, controlled blood lipid, and blood pressure in our patients.

In the same instance, serum irisin levels did not differ significantly between those with a BMI 25 and those with a BMI > 25 (*P* = 0.17), which may have been caused by the non-significant difference in fat mass, which is known to have a direct effect on irisin level. Our results did not concur with Moreno et al. [25], who reported a decrease in irisin levels in obese individuals, nor with De Meneck et al. [26], who reported an increase in serum irisin levels in obese patients. However, we did concur with Icli et al. [18], who reported no significant difference between obese and non-obese BD patients.

The regression analysis of factors affecting CIMT in a patient with Behcet’s disease revealed that the most influential factors on the disease were the patient’s age and ESR level. The patient’s age is one of the risk factors for atherosclerosis and can be determined by CIMT level [27, 28], and BD is an inflammatory disease that increases ESR level.

For characteristics that indicate subclinical atherosclerosis, male gender and decreasing S levels are predictive. Irisin, and decreasing ankle brachial pressure index agreed with Vogt et al. [29], who stated that ABPI ratio of less than 0.9 was associated with up to a threefold relative increase in all-cause and cardiac mortality, and agreed as well with Lee et al. [11] whose results demonstrated a significant correlation between serum irisin level and carotid atherosclerosis in dialysis patients. Seyahi et al. [30] also demonstrated insignificant gender differences.

Icli et al. [18] provided support for irisin as a unique and successful therapeutic target or therapeutic strategy. Chen J et al. [31] concluded that irisin has been shown to reduce nicotine’s role in atherosclerosis and to have antagonistic effects on endothelial cell migration and proliferation. Consequently, irisin has the potential to be used as an anti-atherosclerosis therapy.

We also found a very strong correlation between CIMT, an indicator of subclinical atherosclerosis, and decreased irisin levels in BD patients.

With a sensitivity of 94.30% and specificity of 93.33%, the declining level of S. irisin in patients can be utilized as a predictor of subclinical atherosclerosis. This finding agreed with Ankle Brachial Index Collaboration, 2008 [32], which demonstrated that the ABPI is an independent risk factor for cardiovascular mortality in addition to the traditional Framingham risk factors, and that having an ABPI 0.9 doubled the risk of cardiovascular mortality and morbidity.

## Conclusion

Irisin in the serum is recommended for detecting subclinical atherosclerosis in Behcet’s disease patients.

We recommend that more longitudinal studies of patients be conducted at this point.

Cross-sectional study, small sample size, and low disease activity indexes in our patients are limitations of the study.

## Data Availability

Not available.
